# Facile Synthesis and Herbicidal Evaluation of 4*H*-3,1-Benzoxazin-4-ones and 3*H*-Quinazolin-4-ones with 2-Phenoxymethyl Substituents

**DOI:** 10.3390/molecules17033181

**Published:** 2012-03-14

**Authors:** Zumuretiguli Aibibuli, Yufeng Wang, Haiyang Tu, Xiaoting Huang, Aidong Zhang

**Affiliations:** Key Laboratory of Pesticide and Chemical Biology of Ministry of Education, College of Chemistry, Central China Normal University, Wuhan 430079, China; Email: zumrat010185@yahoo.com (Z.A.); wangyufeng1213@126.com (Y.W.); hxtowen@hotmail.com (X.H.)

**Keywords:** 4*H*-3,1-benzoxazin-4-ones, 3*H*-quinazolin-4-ones, active substructure combination, herbicidal activity, auxinic receptor TIR1

## Abstract

Series of 4*H*-3,1-benzoxazin-4-ones and 3*H*-quinazolin-4-ones with phenoxy-methyl substituents were rationally designed and easily synthesized via one-pot *N*-acylation/ring closure reactions of anthranilic acids with 2-phenoxyacetyl chlorides to yield the 4*H*-3,1-benzoxazin-4-ones, and subsequently substituted with amino derivatives to obtain the 3*H*-quinazolin-4-ones. The herbicidal evaluation was performed on the model plants barnyard grass (a monocotyledon) and rape (a dicotyledon), and most of the title compounds displayed high levels of phytotoxicity. The active substructure and inhibitory phenotype analysis indicated that these compounds could be attributed to the class of plant hormone inhibitors. A docking study of several representative compounds with the hormone receptor TIR1 revealed an appreciable conformational match in the active site, implicating these compounds are potential lead hits targeting this receptor.

## 1. Introduction

Benzoxazinones are an important class of heterocyclic compounds with diverse biological properties that have been widely explored and applied in pharmaceutical and agricultural chemicals. Among benzoxazinones, 4*H*-1,4-benzoxazin-3-one is one of the naturally occurring secondary metabolites of indole [1] and its derivatives have been extensively used for herbicide development [2] and several commercial herbicides such as flumioxazin and thidiazimin contain the core 4*H*-1,4-benzoxazin-3-one structure [3]. Another two subclasses of benzoxazinone derivatives with the 4*H*-3,1-benzoxazin-4-one and 4*H*-1,3-benzoxazin-4-one core structures are also of natural origin and show various promising activities [4]. For example, 4*H*-3,1-benzoxazin-4-ones have been demonstrated to be potent inhibitors of human neutrophil elastase [5]; whereas 4*H*-1,3-benzoxazin-4-ones are potent inhibitors of the acetyl coenzyme A carboxylases (ACCase) of humans, fungi, and plants [6]. The mentioned subclasses of benzoxazinones can be viewed as the bioisosteric forms of one another. More attractive to us is the fact that one 4*H*-3,1-benzoxazin-4-one derivative, namely the 1,1-dimethylethyl ester of α-[(5-methyl-4-oxo-4*H*-3,1-benzoxazin-2-yl)amino]benzeneacetic acid, was identified by screening 500,000 compounds as a potent inhibitor of a new herbicidal target, carboxy terminal processing protease of D1 protein [7].

Synthesis of new benzoxazinones with diverse substituents is a promising practice when searching for potent herbicides, especially based on the core structures of natural metabolites. One important methodology in expanding the molecular structural diversity is the principle of active substructure combination [8], and identification of active substructures is the key step in this purpose. Substituted phenoxyalkyl groups exist in many commercial herbicides and can be envisaged as the key substructure in herbicides ranging from the ACCase inhibitors (aryloxyphenoxypropionates) to the synthetic auxin herbicides (phenoxycarboxylic acids, for example, 2,4-D and 2,4-DB in this case) [9]. Combination of the active 4*H*-3,1-benzoxazin-4-one and substituted phenoxyalkyl fragment substructures is expected to produce novel compounds with desirable bioactivity.

**Scheme 1:**
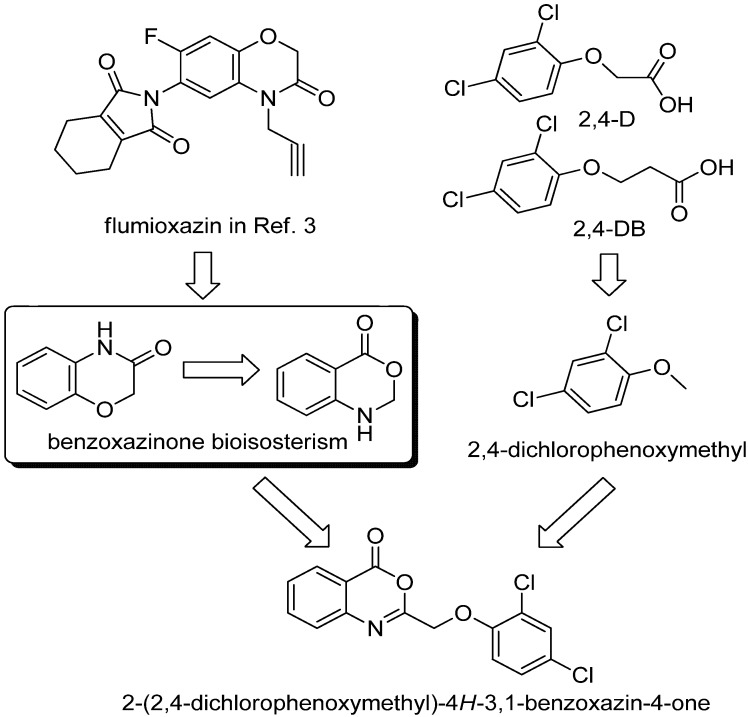
Active substructure analysis and combination for designing the title compounds.

The present work takes advantage of these active substructures and designs a series of novel 2-phenoxymethyl-4*H*-3,1-benzoxazin-4-ones according to the principle of active substructure combination as shown in Scheme 1. The facile synthesis of a series of 2-phenoxymethyl-4*H*-3,1-benzoxazin-4-ones is established. Thanks to the transformable character of the 4*H*-3,1-benzoxazin-4-one core structure, part of synthesized 4*H*-3,1-benzoxazin-4-ones is converted to afford 2-phenoxymethyl-3*H*-quinazolin-4-ones. The structures of all the new compounds were confirmed by ^1^H-NMR, ^13^C-NMR, and MS, and their phytotoxicities evaluated on the model plants including a monocotyledon (barnyard grass) and a dicotyledon (rape). The inhibitory phenotype indicates most of the title compounds can be attributed to the class of hormone type inhibitors. A docking study was performed by docking several representative compounds into the active site of the plant hormone receptor TIR1. The results from the inhibitory phenotype and docking study suggest the synthesized compounds might target the TIR1 receptor.

## 2. Results and Discussion

### 2.1. Chemistry

**Scheme 2:**
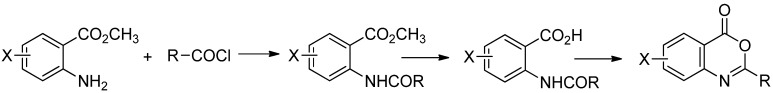
General method for the synthesis of 4*H*-3,1-benzoxazin-4-ones.

4*H*-3,1-Benzoxazin-4-ones are generally synthesized from the starting materials methyl anthranilate and acyl chlorides, as shown in Scheme 2. After formation of the amide linkage, the methyl ester group is hydrolyzed to release the carboxyl group, and then a condensing agent is used to form the fused 4*H*-3,1-benzoxazin-4-one ring [10,11].

We have tried this method according to the literature; unfortunately, the reactions generally gave low yields of the desired products. Since the hydrolysis of the methyl ester group requires catalysis by 6 M HCl, concomitant hydrolysis of the amide linkage is unavoidable in the second step. Another adverse factor is the use of concentrated sulfuric acid or pyridine to promote the cyclization reaction in the third step, which again makes the breakage of the amide linkage possible. All the mentioned reaction conditions tend to result in the low yields of the products in the three-step synthesis. 

**Scheme 3:**
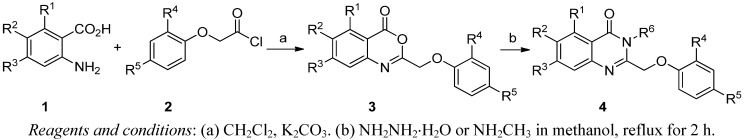
Synthesis of 2-phenoxy-4*H*-3,1-benzoxazin-4-ones (**3**a–w) and 2-phenoxy-3*H*-quinazolin-4-ones (**4**a–s).

In the above synthesis, the reactant methyl anthranilate actually was obtained by esterification of anthranilic acid with methanol; whereas the deprotection of the ester methyl group in the second step is unfavorable for the synthesis. Since a slight reactivity difference exists between the two functional groups carboxylate and amino on the benzene ring in the presence of a base towards acyl chloride, screening for a suitable base in the synthesis without the use of protection and deprotection procedures is possible. Thus, various organic and inorganic bases were tried for this purpose, and fortunately, potassium carbonate was found to be the most favorable base for the synthesis. Without the protection and deprotection procedures for the carboxylic acid group, the cheaply available anthranilic acid can be used directly. More importantly, the *N*-acylation of anthranilic acid with 2-phenoxyacetyl chloride and the subsequent ring closure reaction were found to be accomplished in a single procedure in dichloromethane under the presence of potassium carbonate by stirring at room temperature for no more than 2 hours. After column chromatography on silica gel using petroleum ether/ethyl acetate in the volumetric ratio of 9:1 as the eluent, pure 2-phenoxy-4*H*-3,1-benzoxazin-4-ones were obtained. Thus a convenient and facile one-pot synthesis of various 2-phenoxy-4*H*-3,1-benzoxazin-4-ones by reacting anthranilic acids with 2-phenoxyacetyl chlorides was established (Scheme 3).

The 2-phenoxy-4*H*-3,1-benzoxazin-4-ones can be converted to the corresponding 2-phenoxy-3*H*-quinazolin-4-ones by reacting with various amines via the reported procedure [12]. In this work, hydrazine and methylamine were used. The reaction was accomplished in refluxing ethanol for 3 hours, and generally after cooling to room temperature, the desired product spontaneously precipitated from the reaction mixture in high purity. The syntheses of 2-phenoxy-4*H*-3,1-benzoxazin-4-ones and 2-phenoxy-3*H*-quinazolin-4-ones are outlined in Scheme 3. Thus, 23 2-phenoxy-4*H*-3,1-benzoxazin-4-one compounds and 19 2-phenoxy-3*H*-quinazolin-4-ones compounds were obtained.

**Scheme 4:**
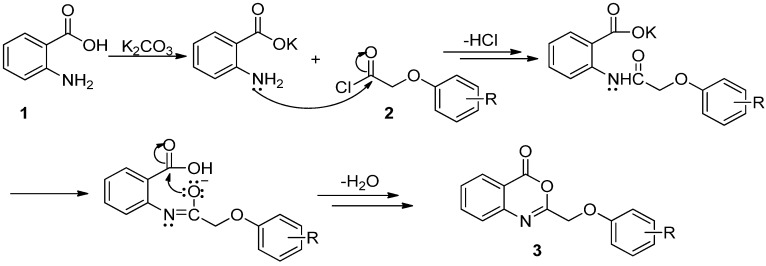
Proposed mechanism for the one-pot synthesis of 2-phenoxy-4*H*-3,1-benzoxazin-4-ones.

A plausible imechanism for the one-pot synthesis of 2-phenoxy-4*H*-3,1-benzoxazin-4-ones from the reaction of anthranilic acids with 2-phenoxyacetyl chlorides in the presence of potassium carbonate is illustrated in Scheme 4. First, anthranilic acid is converted to potassium anthranilate in the presence of the base; with the addition of 2-phenoxyacetyl chloride, the amino group of potassium anthranilate is acylated and potassium *N*-phenoxyacetylanthranilate generated, losing one equivalent of HCl. Through the proton transfer and the resonance of N-C and C-O bonds in the amido group, an iminoxy anion is produced, which in turn attacks the carboxyl group and one molecule of H_2_O is lost, leading to the occurrence of the desired cyclization for the final 2-phenoxy-4*H*-3,1-benzoxazin-4-one product. This process is distinct from the reported one [11], which consists of the stepwise N-acylation, deesterification, and base-promoted cyclization.

The conversion of 2-phenoxy-4*H*-3,1-benzoxazin-4-ones to 2-phenoxy-3*H*-quinazolin-4-ones is a conventional process using hydrazine or alkylamine, in which a combined nucleophilic addition/ring opening and ring-closing/elimination process should be involved. ^1^H-NMR and IR analyses confirm the transformation reaction. For example, in the ^1^H-NMR spectrum of **4**i (the product from the reaction with hydrazine), a conspicuous peak appears at a chemical shift of δ 4.599 ppm, which disappears after adding a drop of D_2_O. In the IR spectrum of **4**i, two peaks at 3,395 cm^−1^ and 1,354 cm^−1^ can be assigned to the absorption bands of the out-ring amino group. These NMR and IR signals cannot be found in the corresponding spectra of the starting material 2-phenoxy-4*H*-3,1-benzoxazin-4-one (**3**p). A similar phenomenon happens when 2-phenoxy-4*H*-3,1-benzoxazin-4-ones react with methylamine.

The structures of all the synthesized 2-phenoxy-4*H*-3,1-benzoxazin-4-ones and 2-phenoxy-3*H*-quinazolin-4-ones have been confirmed by ^1^H-NMR, ^13^C-NMR, IR and MS, and all the data can be found in the Experimental section.

### 2.2. Herbicidal Activity and Inhibition Phenotype

The synthesized compounds were tested for the herbicidal activity on model plants including a monocotyledon (barnyard grass) and a dicotyledon (rape) by the reported Petri dish culture method [13]. The percent inhibitory ratios against the growth of root and stalk of barnyard grass and rape at different dosage concentrations were calculated, and the compounds with appreciable inhibitory potencies are selected and shown in Table 1 and Table 2. Several features of the inhibitory activities can be derived. First, all the compounds have stronger inhibition against the growth of the dicotyledon rape than against the monocotyledon barnyard grass, showing appreciable selectivity. Second, 2-phenoxy-4*H*-3,1-benzoxazin-4-ones have generally higher activities than 2-phenoxy-3*H*-quinazolin-4-ones; Third, within the group of 2-phenoxy-4*H*-3,1-benzoxazin-4-ones, several compounds, including **3**m, **3**o and **3**p have much higher herbicidal activities, comparable to the commercial herbicidal 2,4-D.

**Table 1:** The percent inhibitory ratios against the growth of root and stalk of barnyardgrass and rape at different dosage concentrations of 2-phenoxy-4*H*-3,1-benzoxazin-4-ones **3**.

No.	R^1^	R^2^	R^3^	R^4^	R^5^	Relative inhibition (root/stalk%)			
						Barnyard grass		Rape	
						10 mg/L	1 mg/L	10 mg/L	1 mg/L
**3**a	H	H	H	H	H	64.8/26.9	19.3/−29.6	89.1/79.4	71.2/27
3b	Cl	H	H	H	H	56.3/24.1	47.2/0.5	83.3/51.9	59.6/39.2
3c	H	Cl	H	H	H	62.6/46.7	23.7/0.3	83.9/64.6	45.4/26.7
**3**d	H	H	Cl	H	H	62.3/28.3	41.9/−23.6	93.5/71.6	75.5/44.9
**3**e	H	CH_3_	H	H	H	71.4/31.1	26.1/−17.2	92.3/81.6	55.3/11.6
**3**f	H	OCH_3_	OCH_3_	H	H	63.0/46.7	35.5/32.0	71.1/63.3	94.9/32.9
**3**g	H	H	H	H	Cl	79.5/−1.8	53.7/−25.6	100.0/68.0	98.0/56.8
**3**h	Cl	H	H	H	Cl	88.7/41.9	59.8/14.7	100.0/91.7	96.3/62.0
**3**i	H	Cl	H	H	Cl	87.8/13.2	55.3/−7.3	100.0/82.1	91.4/40.2
**3**j	H	H	Cl	H	Cl	92.3/31.1	43.6/12.8	100.0/91.1	91.4/59.5
**3**k	H	CH_3_	H	H	Cl	77.1/−8.8	48.4/18.2	100.0/82.1	84.7/29.3
**3**l	H	OCH_3_	OCH_3_	H	Cl	78.7/22.0	65.5/12.8	100.0/83.4	96.3/74.5
**3**m	H	H	H	Cl	Cl	100.0/−0.6	38.3/−22.7	100.0/93.8	96.3/77.7
**3**n	Cl	H	H	Cl	Cl	57.5/−1.9	44.4/12.8	79.2/45.9	80.5/38.7
**3**o	H	Cl	H	Cl	Cl	89.4/14.5	73.6/−29.1	100.0/91.4	93.7/66.2
**3**p	H	H	Cl	Cl	Cl	100.0/−14.4	64.3/5.9	100.0/91.4	98.0/82.3
**3**q	H	OCH_3_	OCH_3_	Cl	Cl	51.9/−52.8	35.5/−7.4	100.0/85.2	58.3/14.8
**3**r	H	H	H	H	F	96.8/57.8	62.6/42.8	99.6/87.0	81.4/55.3
**3**s	Cl	H	H	H	F	91.0/46.3	61.1/11.8	98.6/94.8	72.8/25.7
**3**t	H	Cl	H	H	F	93.2/49.1	66.3/36.4	99.3/91.7	85.5/60.0
**3**u	H	H	Cl	H	F	96.7/39.0	61.8/−7.3	99.0/92.2	92.7/65.2
**3**v	H	CH_3_	H	H	F	92.2/31.7	69.9/24.1	99.3/93.7	87.7/61.0
**3**w	H	OCH_3_	OCH_3_	H	F	64.5/16.4	37.5/12.8	79.7/41.4	35.2/12.8
**2,4-D**						99.1/76.2	67.3/45.5	100.0/93.6	91.0/89.1

**Table 2:** The percent inhibitory ratios against the growth of root and stalk of barnyardgrass and rape at different dosage concentrations of 2-phenoxy-3*H*-quinazolin-4-ones **4**.

No.	R^1^	R^2^	R^3^	R^4^	R^5^	R^6^	Relative inhibition (root/stalk%)			
							Barnyard grass		Rape	
							10 mg/L	1 mg/L	10 mg/L	1 mg/L
**4**a	Cl	H	H	H	Cl	NH_2_	43.1/14.9	57.9/8.9	41.5/−5.9	28.5/13.2
**4**b	H	Cl	H	H	Cl	NH_2_	9.9/11.9	33.2/20.9	46.8/13.2	27.7/14.7
**4**c	H	H	Cl	H	Cl	NH_2_	42.1/17.9	10.4/1.4	69.7/41.6	27.7/−9.3
**4**d	H	CH_3_	H	H	Cl	NH_2_	35.6/7.4	16.3/11.9	31.3/-3.4	26.4/−14.7
**4**e	H	OCH_3_	OCH_3_	H	Cl	NH_2_	23.3/11.9	13.8/2.9	30.8/6.4	21.9/1.5
**4**f	H	H	H	Cl	Cl	NH_2_	22.2/−6.6	35.0/−5.0	73.4/48.1	65.6/48.1
**4**g	Cl	H	H	Cl	Cl	NH_2_	25.8/−1.8	11.8/−5.9	17.5/44.9	5.7/10.0
**4**h	H	Cl	H	Cl	Cl	NH_2_	37.5/−12.7	20.0/−42.0	22.0/19.9	7.5/10.7
**4**i	H	H	Cl	Cl	Cl	NH_2_	68.5/38.1	24.3/−7.2	98.2/95.7	93.6/92.3
**4**j	H	OCH_3_	OCH_3_	Cl	Cl	NH_2_	60.0/5.0	7.7/8.3	84.9/67.2	58.4/40.2
**4**k	Cl	H	H	H	Cl	CH_3_	50.9/1.5	32.2/7.5	81.9/52.9	48.8/21.6
**4**l	H	Cl	H	H	Cl	CH_3_	33.7/11.9	24.7/8.9	42.2/10.8	28.5/1.5
**4**m	H	H	Cl	H	Cl	CH_3_	29.2/14.9	14.8/7.5	27.9/−0.5	26.2/1.5
**4**n	H	CH_3_	H	H	Cl	CH_3_	43.5/14.9	26.7/14.9	45.0/16.6	39.4/7.8
**4**o	H	OCH_3_	OCH_3_	H	Cl	CH_3_	17.3/5.9	13.3/8.9	34.8/−6.8	25.1/1.9
**4**p	H	H	H	Cl	Cl	CH_3_	29.4/−47.3	2.5/−10.9	21.2/29.2	4.5/27.1
**4**q	H	Cl	H	Cl	Cl	CH_3_	31.9/−3.6	24.3/5.4	19.3/38.9	8.1/14.4
**4**r	H	H	Cl	Cl	Cl	CH_3_	34.6/0.0	12.3/1.4	44.5/−2.4	32.3/−8.8
**4**s	H	OCH_3_	OCH_3_	Cl	Cl	CH_3_	52.2/−54.5	30.0/−20.3	20.7/20.3	7.1/8.1
**2,4-d**							99.1/76.2	67.3/45.5	100.0/93.6	91.0/89.1

Among the title compounds, 2-phenoxy-4*H*-3,1-benzoxazin-4-ones **3** have good to excellent herbicidal activities against the root growth of rape, even at a concentration down to subnanomolar levels for **3**m, **3**o and **3**p. However, 2-phenoxy-3*H*-quinazolin-4-ones **4** generally show comparatively lower activities. The herbicidal activities of these new compounds vary with the type and position of substituents on both the aromatic rings of benzoxazinone and phenoxymethyl group. Generally, electron-withdrawing substituents, such as chloro and fluoro, can give rise to a high activity; whereas electron-releasing groups, such as methoxy or methyl on the ring, obviously decrease the activity. For example, benzoxazinones with the dichloro substitution (**3**m to **3**q) are found to possess the high herbicidal activities. On the other hand, monochloro substitution (**3**g to **3**l) decreases the activity notably, and monofluoro substitution (**3**r to **3**w) decreases the activity even further. The lowest activity can be found for the compounds with no substituent on the phenoxymethyl group (**3**a to **3**f). The octanol-water partition coefficient logP values were calculated, and most of the values are found to be in the region of 3–4 for 2-phenoxy-4*H*-3,1-benzoxazin-4-ones **3** and 2-phenoxy-3*H*-quinazolin-4-ones **4**. For this reason, there is no distinct difference in the herbicidal activities.

Several representative compounds selected from the 2-phenoxy-4*H*-3,1-benzoxazin-4-one and 2-phenoxy-3*H*-quinazolin-4-one groups were tested their concentration dependant activities and the half maximal inhibitory concentrations (IC_50_ values) are shown in Table 3. Obviously, the 2-phenoxy-4*H*-3,1-benzoxazin-4-ones **3**m and **3**o have IC_50_ values near to that of the commercial herbicidal 2,4-D. More importantly, these molecules consist of 2-phenoxymethyl group with halo substituents both at the 2- and 4- positions of the benzene ring, a similar pattern to the commercial hormone herbicides 2,4-D, clomeprop, and 2,4-DB, as well as the ACCase herbicide diclofop-methyl. This result implicates the halo groups at both 2- and 4- positions on the benzene ring plus the benzene ring itself offer another powerful active substructure in the highly active compounds. Moreover, if no halo group exists on the benzene ring, the activity will decrease dramatically. On the other hand, the substructure 4*H*-3,1-benzoxazin-4-one in this work and the carboxylic group in the commercial phenoxyethanoic and phenoxypropionic acid herbicides play also a crucial role in the contribution to the activities of the compounds, meaning they are active substructures. A control experiment has been conducted by using 2,4-dichlorophenyl ethyl ether, a compound without the 4*H*-3,1-benzoxazin-4-one substructure mentioned here, which loses nearly all of its herbicidal activity.

**Figure 1:**
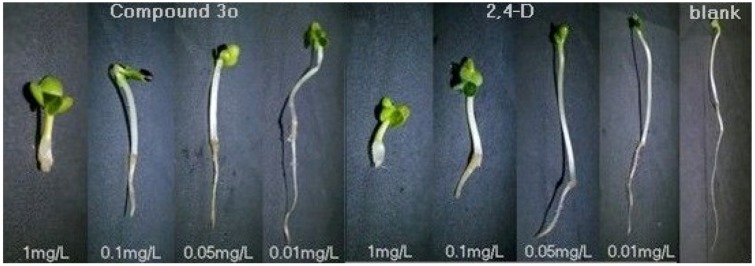
Photographs showing the lateral root development of dicotyledon rape at different concentrations of **3o** (the first four specimens), 2,4-D(the second four specimens), and the control (the last specimen), respectively. Photographs were taken after 7 days treatment.

Besides the features of the active 4*H*-3,1-benzoxazin-4-one and 2,4-dichloro phenoxyalkyl group substructures in the synthesized compounds, another distinct one is that almost all of them showed a inhibition phenotype similar to that of the hormone type herbicide 2,4-D. This type of inhibition is characterized by the abnormal growth of the deformed shoots, a breakdown of chlorophyll, and the disruption of the root elongation, while this abnormal growth leads to the death of the plant. The inhibition phenotype of the typical compound **3**o against the growth of dicotyledon rape was exemplarily photographed and shown in Figure 1 (the first four specimens). The controls obtained from the inhibition by 2,4-D (the second four specimen in Figure 1) and blank (the last specimen in Figure 1) are also shown for comparison. 

**Table 3:** IC_50_ values for the root elongation inhibition of rape for selected compounds from **3** and **4**.

No.	R^1^	R^2^	R^3^	R^4^	R^5^	R^6^	IC_50_ (μmol)
**3**g	H	H	H	H	Cl		30.37
**3**h	Cl	H	H	H	Cl		138.24
**3**i	H	Cl	H	H	Cl		22.32
**3**l	H	OCH_3_	OCH_3_	H	Cl		80.14
**3**m	H	H	H	Cl	Cl		10.34
**3**n	Cl	H	H	Cl	Cl		43.54
**3**o	H	Cl	H	Cl	Cl		10.73
**3**p	H	H	Cl	Cl	Cl		11.05
**3**q	H	OCH_3_	OCH_3_	Cl	Cl		142.22
**4**f	H	H	H	Cl	Cl	NH_2_	224.37
**4**i	H	H	Cl	Cl	Cl	NH_2_	27.23
**4**j	H	OCH_3_	OCH_3_	Cl	Cl	NH_2_	167.59
**4**k	Cl	H	H	H	Cl	CH_3_	201.13
**2,4-D**							6.06

Obviously, both the compound **3**o and 2,4-D show a similar inhibition phenotype, *i.e.*, dwarf stalks, leaf chlorosis and disruption of the root elongation with an obvious concentration dependant relationship. This kind of phenotype is well known and attributed to the class of hormone herbicide symptoms. Together with the consideration of similar active substructures involved in both 4*H*-3,1-benzoxazin-4-ones and phenoxymethyl carboxylate herbicides, there is no wonder that all of the synthesized compounds may be regarded to target a similar site as does the well known herbicide 2,4-D.

### 2.3. Docking Study

The active substructure analysis and the inhibitory phenotype implicate that most of the title compounds could be attributed to the class of hormone type inhibitors. 2,4-D is a typical hormone type inhibitor and its action site is the auxinic receptor TIR1, which has been well documented in recent years [14]. The synthesized compounds in this work may attack this protein target like 2,4-D according to the above analyses based on the active substructures and the herbicidal phenotype. Thus a molcular docking study was performed by docking of several representative compounds into the active site of the plant hormone receptor TIR1, using the autodock software Vina according to the introduction of the docking software designer Dr. Oleg Trott in the Molecular Graphics Lab at the Scripps Research Institute [15]. The visualization and comparison of the docking results were realized using the tool MGLTools 1.5.4.

**Figure 2:**
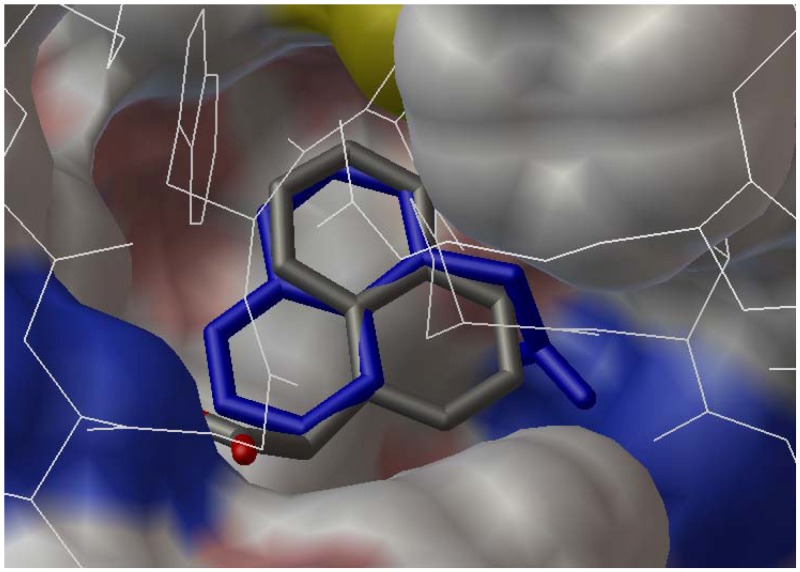
Superimposed conformation of the docked naphthalen-1-yl acetic acid (in atomic color) with its crystal (in blue) in the complex TIR1/IAA (PDB ID 2P1O).

The complex crystal structures of the auxinic receptor TIR1 with small molecule agonists and antagonists have been reported, including 2,4-D and naphthalen-1-yl acetic acid (NAA) [16], as well as α-alkyl indole-3-acetic acids [17]. X-ray crystallography shows that these small molecules bind at the bottom of the TIR1 pocket with an unexpected co-factor inositol hexakisphosphate nearby the binding site, and above the binding small molecule is an Aux/IAA substrate peptide [16,17]. Therefore, in our docking experiments, the complex crystal data of TIR1/NAA (PDB ID: 2P1O) was chosen and the receptor file was prepared by extracting all water molecules and the bound ligand NAA from the crystal data. The ligand files of the selected compounds were prepared using the conformational energy minimization. The compiled config file was executed in Vina and the docked conformation with the lowest binding energy was chosen for the comparison. In order to validate the credibility of the docking procedure, NAA was docked and superimposed with its crystal conformation in the binding site of TIR1 receptor (Figure 2). It reveals both of the conformations overlap fairly well, especially for the portion of naphthalenyl moiety in the molecule, demonstrating the acceptable accuracy by using the docking parameters in Vina software.

**Figure 3:**
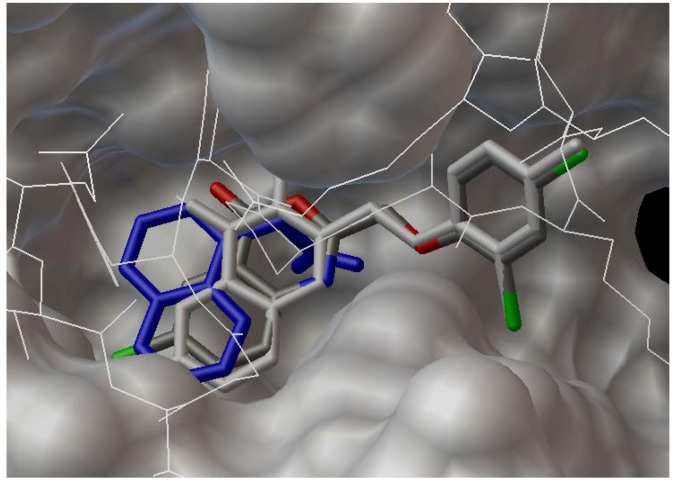
A top view of the docked conformations of **3**o (in atomic color) and **4**i (in grey) along with NAA (in blue) from the TIR1/NAA crystal complex in the binding site of the receptor TIR1. The above line structure is the Aux/IAA substrate peptide.

The selected representative compounds **3**o and **4**i were docked into the active site of the above prepared receptor using the same procedure mentioned, and the stacked conformations in the active site are shown in Figure 3. The compounds (**3**o: Atomic color; **4**i: Grey color) are well resided in the active site using the conformation of NAA (blue color) from the crystal structure of TIR1/NAA as the indicator. The Aux/IAA substrate peptide in line formula is also shown above the docked molecules. The 4*H*-3,1-benzoxazin-4-one moiety from **3**o and 3*H*-quinazolin-4-one from **4**i stack very well with the naphthalene ring from NAA, meaning the full occupation of the active site space. On the other hand, the 2,4-dichlorophenoxy moiety from **3**o and **4**i directs away from the active site, a common orientation observed for the alkyl group in the complexes of TIR1/α-alkyl indole-3-acetic acids [17]. 

The predicted binding affinities for **3**o and **4**i to the receptor are −9.6 and −8.6 kcal/mol, respectively. The tendency of the predicted binding affinities is consistent with inhibitory efficacies of **3**o and **4**i against the rape root growth, which are 10.37 and 20.23 nM, respectively, in IC_50_ measurements. The difference between the binding affinity and inhibitory efficacy may come from the disturbances of ring electron density and steric hindrance of the substituent on the ring member nitrogen of 3*H*-quinazolin-4-one. On the other hand, the predicted binding energy for 2,4-D is only −6.8 nM, whereas its inhibitory efficacy IC_50_ reaches down to 6.06 nM. The higher binding affinity but lower IC_50_ value for 2,4-D as compared with **3**o or **4**i reflects that other factors such as absorption, transportation, *etc.*, may deteriorate the inhibitory efficacies of **3**o or **4**i. Thus, further future structural optimization of 2-phenoxy-4*H*-3,1-benzoxazin-4-ones and 2-phenoxy-3*H*-quinazolin-4-ones should be focused on improving their absorption and transportation by target plants functions.

## 3. Experimental

### 3.1. General

All solvents were redistilled before use. Melting points were taken on a Buchi B-545 melting point apparatus and the temperatures are uncorrected. ^1^H-NMR and ^13^C-NMR spectra were recorded on a Mercury-Plus 400 or Mercury-Plus 600 spectrometer in CDCl_3_ using TMS as an internal reference. IR spectra were recorded on a Nicolet 360 infrared spectrometer as KBr pellets with absorption in cm^−1^. MS were measured on a Finnigan Trace MS spectrometer, at 70 eV. Unless otherwise noted, all starting materials are commercially available and were used directly without further purification.

### 3.2. Synthesis

*Step 1. Synthesis of phenoxyacetic* *chlorides.* Phenoxyacetic chlorides were synthesized according to the reported method [18]. Briefly, the mixture of phenoxyacetic acid (10 mmol) and thionyl chloride (6–10 mL) was reacted for 3 h under reflux until no further gaseous HCl was released. Excess thionyl chloride was distilled off under reduced pressure, giving the corresponding phenoxyacetic chlorides (~98% yield) as brown oils. The phenoxyacetic chlorides were used in the next step without further purification.

*Step 2. General synthesis of 2-phenoxymethyl-4H-3,1-benzoxazin-4-ones* **3a**–**w**. In a 250-mL flask, anthranilic acid (10 mmol) and anhydrous potassium carbonate (20 mmol) were mixed in dry CH_2_Cl_2_ (100 mL) under stirring in an ice bath, then phenoxyacetic chloride (15 mmol) in CH_2_Cl_2_ (10 mL) was added dropwise. After the addition, the solution was stirred for 2 h at room temperature. The mixture was filtered and washed with CH_2_Cl_2_. The filtrate was washed with saturated brine and the organic layer was dried with sodium sulfate. After evaporating the solvent the residue was chromatographed on silica gel using the mixed solvents of ether/petroleum ether (volumetric ratio: 1:9). Molecular structures, melting points and yields are summarized in Table 4.

**Table 4:** Molecular structures, yields, and melting points of synthesized 2-phenoxy-4*H*-3,1-benzoxazin-4-ones **3**.

No.	R^1^	R^2^	R^3^	R^4^	R^5^	Appearance	Mp/°C	Yield/%
**3**a	H	H	H	H	H	pale yellow solid	87.9~92.1	85.6
**3**b	Cl	H	H	H	H	white solid	118.3~120.9	87.1
**3**c	H	Cl	H	H	H	pale yellow solid	113.1~115.2	79.8
**3**d	H	H	Cl	H	H	pale yellow solid	117.3~119.9	87.1
**3**e	H	CH_3_	H	H	H	pink solid	133.3~135.1	85.2
**3**f	H	OCH_3_	OCH_3_	H	H	pale yellow solid	132.1~134.3	76.1
**3**g	H	H	H	H	Cl	white solid	147.5~149.2	89.1
**3**h	Cl	H	H	H	Cl	white solid	144.3~145.7	80.1
**3**i	H	Cl	H	H	Cl	pale yellow solid	153.2~154.6	79.5
**3**j	H	H	Cl	H	Cl	pale yellow solid	189.9~191.8	83.3
**3**k	H	CH_3_	H	H	Cl	pale yellow solid	143.7~145.6	92.3
**3**l	H	OCH_3_	OCH_3_	H	Cl	white solid	118.5~119.3	90.8
**3**m	H	H	H	Cl	Cl	white solid	118.5~119.3	73.5
**3**n	Cl	H	H	Cl	Cl	white solid	161.9~163.2	81.1
**3**o	H	Cl	H	Cl	Cl	white solid	157.9~160.1	78.6
**3**p	H	H	Cl	Cl	Cl	white solid	139.6~142.5	88.1
**3**q	H	OCH_3_	OCH_3_	Cl	Cl	white solid	161.9~163.2	90.2
**3**r	H	H	H	H	F	white solid	112.5~114.6	92.2
**3**s	Cl	H	H	H	F	white solid	155.9~156.7	77.3
**3**t	H	Cl	H	H	F	white solid	159.8~161.7	87.1
**3**u	H	H	Cl	H	F	white solid	123.6~124.9	89.9
**3**v	H	CH_3_	H	H	F	white solid	130.8~131.9	91.4
**3**w	H	OCH_3_	OCH_3_	H	F	white solid	144.7~146	96.2

*2-(Phenoxymethyl)-4*H*-3,1-benzoxazin-4-one* (**3a**). ^1^H-NMR (400 MHz, CDCl_3_): δ 5.018 (s, 2H, -CH_2_O-), 7.028 (d, *J* = 17.2 Hz, 3H, Ar-H), 7.303~7.341 (m, 2H, Ar-H), 7.550~7.587 (m, 1H, Ar-H), 7.668 (d, *J* = 8.4 Hz, 1H, Ar-H), 7.819~7.857 (m, 1H, Ar-H), 8.227 (d, *J* = 8 Hz, 1H, Ar-H). IR (KBr) ν: 3020,1763, 1695, 1606, 1496, 1243, 760 cm^−1^. EI-MS *m/z* (%): 253.04 (M^+^, 44.47), 77.03 (53.73), 132.05 (61.88), 253.04 (44.74), 145.97 (100.00).

*5-Chloro-2-(phenoxymethyl)-4*H*-3,1-benzoxazin-4-one* (**3b**). ^1^H-NMR (400 MHz, CDCl_3_): δ 4.99 (s, 2H, -CH_2_O-), 7.22 (d, *J* = 6.8 Hz, 3H, Ar-H), 7.303~7.321 (m, 2H, Ar-H), 7.571 (d, *J* = 4 Hz, 2H, Ar-H), 7.696 (d, *J* = 4 Hz, 1H, Ar-H). IR (KBr) ν: 3023, 1751, 1659, 1587, 1492, 1230, 787 cm^−1^. EI-MS *m/z* (%): 286.92 (M^+^, 23.63), 77.09 (27.30), 181.97 (32.70), 179.91 (100.00).

*6-Chloro-2-(phenoxymethyl)-4*H*-3,1-benzoxazin-4-one* (**3c**). ^1^H-NMR (400 MHz, CDCl_3_): δ 5.011 (s, 2H, -CH_2_O-), 7.027 (d, *J* = 8 Hz, 3H, Ar-H), 7.306~7.344 (m, 2H, Ar-H), 7.411 (d, *J* = 4.1 Hz, 1H, Ar-H), 7.632 (d, *J* = 4 Hz, 1H, Ar-H), 8.188 (d, *J* = 3.6 Hz, 1H, Ar-H). IR (KBr) ν: 3009, 1780, 1671, 1516, 1232, 748 cm^−1^. EI-MS *m/z* (%): 28.99 (M^+^, 39.33), 77.08 (37.04), 179.97 (39.62), 166.01 (100.00).

*7-Chloro-2-(phenoxymethyl)-4*H*-3,1-benzoxazin-4-one* (**3d**). ^1^H-NMR (400 MHz, CDCl_3_): δ 5.010 (s, 2H, -CH_2_O-), 7.021 (d, *J* = 6.4 Hz, 3H, Ar-H), 7.303~7.342 (m, 2H, Ar-H), 7.516 (d, *J* = 8.8 Hz, 1H, Ar-H), 7.660 (s, 1H, Ar-H), 8.146 (d, *J* = 8.4 Hz, 1H, Ar-H). IR (KBr) ν: 2924, 1673, 1602, 1515, 1241, 850 cm^−1^. EI-MS *m/z* (%): 286.94 (M^+^, 32.76), 77.05 (32.95), 123.96 (28.54), 179.93 (100.00).

*6-Methyl-2-(phenoxymethyl)-4*H*-3,1-benzoxazin-4-one *(**3e**). ^1^H-NMR (400 MHz, CDCl_3_): δ 2.391 (s, 3H, -CH_3_), 5.000 (s, 2H, -CH_2_O-), 7.035 (d, *J* = 8.4 Hz, 3H, Ar-H), 7.296~7.335 (m, 2H, Ar-H), 7.560 (d, *J* = 8 Hz, 1H, Ar-H), 7.629 (s, 1H, Ar-H), 8.016 (s, 1H, Ar-H). IR (KBr) ν: 3021, 2947, 1751, 1657, 1585, 1492, 1206, 787 cm^−1^. EI-MS *m/z* (%): 267.01 (M^+^, 34.95), 77.04 (41.69), 160.03 (41.17), 146.00 (100.00).

*6,7-Dimethoxy-2-(phenoxymethyl)-4*H*-3,1-benzoxazin-4-one* (**3f**). ^1^H-NMR (400 MHz, CDCl_3_): δ 3.995 (d, *J* = 3.2 Hz, 6H, -OCH_3_), 5.000 (s, 2H, -CH_2_O-), 7.036 (d, *J* = 8 Hz, 3H, Ar-H), 7.089 (s, 1H, Ar-H), 7.342~7.271 (m, 2H, Ar-H), 7.531 (s, 1H, Ar-H). IR (KBr) ν: 2947, 2873, 1751, 1659, 1587, 1492, 1222, 755 cm^−1^. EI-MS *m/z* (%): 313.11 (M^+^, 25.99), 220.07 (41.27), 192.12 (100.00).

*2-((4-Chlorophenoxy)methyl)-4*H*-3,1-benzoxazin-4-one *(**3g**). ^1^H-NMR (400 MHz, CDCl_3_): δ 4.986 (s, 2H, -OCH_2_-), 6.973 (d, *J* = 9.6 Hz, 2H, Ar-H), 7.264 (d, *J* = 16 Hz, 2H, Ar-H), 7.577 (d, *J* = 16.8 Hz, 1H, Ar-H), 7.645~7.664 (m, 1H, Ar-H), 7.823~8.65 (m, 1H, Ar-H), 8.228 (d, *J* = 9.2 Hz, 1H, Ar-H). IR (KBr) ν: 2932, 1671, 1582, 1514, 1494, 1293, 753 cm^−1^. EI-MS *m/z* (%): 286.97 (M^+^, 32.55), 77.05 (34.92), 90.10( 38.11), 132.05 (95.12), 146.02 (100.00).

*5-Chloro-2-((4-chlorophenoxy)methyl)-4*H*-3,1-benzoxazin-4-one *(**3h**). ^1^H-NMR (400 MHz, CDCl_3_): δ 4.591 (s, 2H, -CH_2_O-), 6.953 (d, 8.8 Hz, 2H, Ar-H), 7.216 (d, *J* = 8.4 Hz, 1H, Ar-H), 7.296~7.389 (m, 3H, Ar-H), 8.330 (s, 1H, Ar-H). ^13^C-NMR (CDCl3) δ: 68.49, 116.59, 119.04, 125.55, 126.99, 129.11, 129.44, 131.64, 136.38, 142.11, 156.22, 166.19, 170.38. IR (KBr) ν: 2952, 1673, 1580, 1515, 1495, 1241, 749 cm^−1^. EI-MS *m/z* (%): 321.82 (M^+^, 5.60), 166.05 (55.61), 240.07 (25.21), 180.03 (100.00).

*6-Chloro-2-((4-chlorophenoxy)methyl)-4*H*-3,1-benzoxazin-4-one *(**3i**). ^1^H-NMR (400 MHz, CDCl_3_): δ 4.976 (s, 2H, -OCH_2_-), 6.96 (d, *J* = 8.8 Hz, 2H, Ar-H), 7.273 (d, *J* = 8.4 Hz, 2H, Ar-H), 7.603 (d, *J* = 8.4 Hz, 1H, Ar-H), 7.777 (d, *J* = 10.8 Hz, 1H, Ar-H), 8.182~8.188 (s, 1H, Ar-H). IR (KBr) ν: 2987, 1697, 1582, 1520, 1493, 1242, 755 cm^−1^. EI-MS *m/z* (%): 320.90 (M^+^, 25.82), 110.97 (27.73), 179.94 (22.04), 165.95 (100.00).

*7-Chloro-2-((4-chlorophenoxy)methyl)-4*H*-3,1-benzoxazin-4-one* (**3j**). ^1^H-NMR (400 MHz, CDCl_3_): δ 4.980 (s, 2H, -CH_2_O-), 6.953 (d, 8.8 Hz, 2H, Ar-H), 6.957 (d, *J* = 8.8 Hz, 2H, Ar-H), 7.261 (d, *J* = 17.2 Hz, 2H, Ar-H), 7.422 (d, *J* = 5.6 Hz, 2H, Ar-H), 7.651 (s, 1H, Ar-H), 8.112 (d, *J* = 4.4 Hz, 2H, Ar-H). ^13^C-NMR (CDCl3) δ: 67.40, 104.22, 114.94, 116.54, 116.69, 123.80, 125.47, 129.26, 129.39, 132.96, 138.63, 141.08, 155.83, 159.06, 167.29. IR (KBr) ν: 2933, 1760, 1665, 1518, 1445, 1280, 817 cm^−1^. EI-MS *m/z* (%): 320.98 (M^+^, 32.11), 123.99 (28.18), 166.00 (58.01), 179.89 (100.00).

*2-((4-Chlorophenoxy)methyl)-6-methyl-4*H*-3,1-benzoxazin-4-one *(**3k**). ^1^H-NMR (400 MHz, CDCl_3_): δ 2.380 (s, 3H, -CH_3_), 4.966 (s, 2H,-OCH_2_-), 6.966 (d, *J* = 8.4 Hz, 2H, Ar-H), 7.262 (d, *J* = 8.8 Hz, 2H, Ar-H), 7.543 (d, *J* = 6.8 Hz, 1H, Ar-H), 7.633 (d, *J* = 2.4 Hz, 1H, Ar-H), 8.017 (s, 1H, Ar-H). ^13^C-NMR (CDCl3) δ: 21.26, 66.74, 104.11, 116.29, 116.88, 126.91, 128.24, 129.48, 137.88, 139.66, 143.38, 148.23, 156.39, 158.77. IR (KBr) ν: 2973, 2945, 1749, 1662, 1618, 1492, 1247, 820 cm^−^^1^. EI-MS *m/z* (%): 301.04 (M^+^, 28.66), 128.14 (32.95), 171.08 (25.40), 146.06 (100.00).

*2-((4-Chlorophenoxy)methyl)-6,7-dimethoxy-4*H*-3,1-benzoxazin-4-one *(**3l**). ^1^H-NMR (400 MHz, CDCl_3_): δ 3.994 (d, *J* = 4 Hz, 6H, -OCH_3_), 4.967 (s, 2H, -OCH_2_-), 6.967 (d, *J* = 9.2 Hz, 2H, Ar-H), 7.072 (s, 1H, Ar-H), 7.279 (d, *J* = 4 Hz, 2H, Ar-H), 7.528 (s, 1H, Ar-H). ^13^C-NMR (CDCl3) δ: 56.43, 56.52, 66.67, 107.55, 108.15, 109.72, 116.29, 126.94, 129.51, 141.91, 150.13, 156.53, 158.56. IR (KBr) ν: 2972, 2847, 1749, 1661, 1615, 1492, 1242, 833 cm^−1^. EI-MS *m/z* (%): 346.95 (M^+^, 10.22), 220.01 (46.59), 192.01 (100.00).

*2-((2,4-Dichlorophenoxy)methyl)-4*H*-3,1-benzoxazin-4-one *(**3m**)*.* ^1^H-NMR (400 MHz, CDCl_3_): δ 5.055 (s, 2H, -OCH_2_-), 7.012 (d, *J* = 8.8 Hz, 1H, Ar-H), 7.187 (d, *J* = 10 Hz, 1H, Ar-H), 7.419 (s, 1H, Ar-H), 7.590 (d, *J* = 7.2 Hz, 1H, Ar-H), 7.639~7.657 (m, 1H, Ar-H), 7.825~7.846 (m, 1H, Ar-H), 8.231~8.240 (s, *J* = 7.2 Hz, 1H, Ar-H). ^13^C-NMR (CDCl3) δ: 67.95, 104.67, 15.72, 124.71, 127.25, 127.69, 128.72, 129.23, 130.42, 136.77, 142.12, 139.81, 152.49, 156.71. IR (KBr) ν: 3039, 1773, 1654, 1591, 1477, 1237, 773 cm^−1^. EI-MS *m/z* (%): 321 (M^+^, 100.00), 119.08 (38.56), 132.04 (42.89), 160.98 (67.85).

*5-Chloro-2-((2,4-dichlorophenoxy)methyl)-4*H*-benzo[3,1]oxazin-4-one* (**3n**). ^1^H-NMR (400 MHz, CDCl_3_): δ 5.055 (s, 2H, -OCH_2_-), 7.012 (d, *J* = 8.8 Hz, 1H, Ar-H), 7.187 (d, *J* = 10 Hz, 1H, Ar-H), 7.419 (s, 1H, Ar-H), 7.590 (d, *J* = 7.2 Hz, 1H, Ar-H), 7.639~7.657 (m, 1H, Ar-H), 7.825~7.846 (m, 1H, Ar-H), 8.231~8.240 (s, *J* = 7.2 Hz, 1H, Ar-H). IR (KBr) ν: 2924, 1752, 1659, 1579, 1477, 1243, 819 cm^−1^. EI-MS *m/z* (%): 355.86 (M^+^, 100.00), 152.98 (47.48), 166.02 (67.72), 179.96 (48.40).

*6-Chloro-2-((2,4-dichlorophenoxy)methyl)-4*H*-3,1-benzoxazin-4-one* (**3o**). ^1^H-NMR (400 MHz, CDCl_3_): δ 5.043 (s, 2H, -OCH_2_-), 6.984 (d, *J* = 8.4 Hz, 1H, Ar-H), 7.175 (d, *J* = 2.4 Hz,1H, Ar-H), 7.422 (s, 1H, Ar-H), 7.597 (d, *J* = 8.4 Hz, 1H, Ar-H), 7.764~7.791 (m, 1H, Ar-H), 8.182~8.188 (s, 1H, Ar-H). ^13^C-NMR (CDCl3) δ: 67.67, 118.19, 118.33, 122.47, 124.59, 127.67, 128.04, 130.41, 134.97, 137.04, 143.91, 152.29, 156.87, 157.38. IR (KBr) ν: 3068 cm^−1^, 1781 cm^−1^, 1689 cm^−1^, 1474 cm^−1^, 1283 cm^−1^, 794 cm^−1^. EI-MS *m/z*(%): 354.96 (M^+^, 9.17), 168.1 (37.70), 319.91 (46.05), 166.08 (100.00).

*7-Chloro-2-((2,4-dichlorophenoxy)methyl)-4*H*-3,1-benzoxazin-4-one *(**3p**). ^1^H-NMR (400 MHz, CDCl_3_): δ 5.047 (s, 2H, -OCH_2_-), 6.982 (d, *J* = 6 Hz, 1H, Ar-H), 7.175~7.258 (m, 2H, Ar-H), 7.424 (s, 1H, Ar-H), 7.539 (d, *J* = 8.4 Hz, 1H, Ar-H), 7.645 (s, 1H, Ar-H), 8.153 (d, *J* = 8.4 Hz, 1H, Ar-H). IR (KBr) ν: 2925, 1736, 1660, 1579, 1492, 1245, 802 cm^−1^. EI-MS *m/z* (%): 355.96 (M^+^, 14.59), 179.98 (78.12), 319.87 (93.89), 166.05 (100.00).

*2-((2,4-Dichlorophenoxy)methyl)-6,7-dimethoxy-4*H*-3,1-benzoxazin-4-one *(**3q**). ^1^H-NMR (400 MHz, CDCl_3_): δ 3.999 (d, *J* = 5.2 Hz, 6H, -OCH_3_), 5.031 (s, 2H, -OCH_2_-), 7.003 (s, 1H, Ar-H), 7.055 (s, 1H, Ar-H), 7.415 (s, 1H, Ar-H), 7.421 (s, 1H, Ar-H), 7.528 (s, 1H, Ar-H). ^13^C-NMR (CDCl3) δ: 56.39, 56.50, 67.81, 107.49, 108.13, 109.69, 115.59, 124.33, 127.38, 127.63, 130.29, 141.79, 150.21, 152.46, 155.89, 156.41, 158.42. IR (KBr) ν: 2927, 2803, 1736, 1651, 1580, 1492, 1247, 803 cm^−1^. EI-MS *m/z* (%): 381.98 (M^+^, 89.40), 191.99 (91.64), 206.03 (78.72), 383.89 (51.06), 221.02 (100.00).

*2-((4-Fluorophenoxy)methyl)-4*H*-3,1-benzoxazin-4-one* (**3r**). ^1^H-NMR (400 MHz, CDCl_3_): δ 4.9796 (s, 2H, -OCH_2_-), 6.971 (d, *J* = 8.6 Hz, 2H, Ar-H), 7.156 (d, *J* = 8 Hz, 2H, Ar-H), 7.560~7.596 (m, 1H, Ar-H), 7.664 (d, *J* = 8.4 Hz, 1H, Ar-H), 7.825~7.845 (m, 1H, Ar-H), 8.229 (d, *J* = 8.8 Hz, 1H, Ar-H). IR (KBr) ν: 2923, 1757, 1693, 1505, 1453, 1273, 787 cm^−1^. EI-MS *m/z* (%): 271.02 (M^+^, 53.36), 90.11 (31.46), 145.98 (83.03), 132.06 (100.00).

**5***-Chloro-2-((4-fluorophenoxy)methyl)-4*H*-3,1-benzoxazin-4-one* (**3s**). ^1^H-NMR (600 MHz, CDCl_3_): δ 4.945 (s, 2H, -CH_2_O-), 6.983 (d, *J* = 2.4 Hz, 2H, Ar-H), 6.995 (d, *J* = 8.4 Hz, 2H, Ar-H), 7.548~7.577 (m, 2H, Ar-H), 7.686 (s, 1H, Ar-H). IR (KBr) ν: 2923, 1756, 1694, 1583, 1505, 1272, 892 cm^−1^. EI-MS *m/z* (%): 305.02 (M^+^, 25.81), 124.04 (17.28), 166.00 (25.57), 181.99 (30.12), 179.95 (100.00).

*6-Chloro-2-((4-fluorophenoxy)methyl)-4*H*-3,1-benzoxazin-4-one* (**3t**). ^1^H-NMR (600 MHz, CDCl_3_): δ 4.963 (s, 2H, -OCH_2_-), 6.5 (d, *J* = 4.2 Hz, 2H, Ar-H), 7.010 (d, *J* = 9.6 Hz, 2H, Ar-H), 7.609 (d, *J* = 9 Hz, 1H, Ar-H), 7.872 (d, *J* = 9 Hz, 1H, Ar-H), 8.183 (s, 1H, Ar-H). IR (KBr) ν: 2923, 1747, 1660, 1606, 1504, 1211, 830 cm^−1^. EI-MS *m/z* (%): 305.00 (M^+^, 28.86), 124.05 (21.22), 168.05 (31.99), 166.01 (100.00).

*7-Chloro-2-((4-fluorophenoxy)methyl)-4*H*-3,1-benzoxazin-4-one* (**3u**). ^1^H-NMR (600 MHz, CDCl_3_): δ 4.964 (s, 2H, -CH_2_O-), 6.985 (d, *J* = 4.8 Hz, 2H, Ar-H), 6.997 (d, *J* = 7.2 Hz, 2H, Ar-H), 7.533 (d, *J* = 4.8 Hz, 1H, Ar-H), 7.656 (s, 1H, Ar-H), 8.147 (d, *J* = 8.4 Hz, 1H, Ar-H). IR (KBr) ν: 2923, 1671, 1661, 1509, 1437, 1223, 772 cm^−1^. EI-MS *m/z* (%): 305.00 (M^+^, 38.01), 124.03 (27.72), 166.03 (51.89), 305.00 (38.01), 180.00 (100.00).

*2-((4-Fluorophenoxy)methyl)-6-methyl-4*H*-3,1-benzoxazin-4-one* (**3v**)*.* ^1^H-NMR (400 MHz, CDCl_3_): δ 2.492 (s, 3H, -CH_3_), 4.960 (s, 2H, -OCH_2_-), 6.986 (d, *J* = 1.2 Hz, 2H, Ar-H), 7.001 (d, *J* = 1.2 Hz, 2H, Ar-H), 7.556 (d, *J* = 7.6 Hz, 1H, Ar-H), 7.647 (d, *J* = 8.4 Hz, 1H, Ar-H), 8.017 (s, 1H, Ar-H). ^13^C-NMR (CDCl_3_) δ: 66.95, 115.51, 115.93, 116.12, 116.16, 116.20, 126.99, 129.61, 129.90, 143.17, 146.56, 153.75, 156.70, 157.78, 158.82, 159.09. IR (KBr) ν: 2961, 2923, 1671, 1509, 1437, 1223, 772 cm^−1^. EI-MS *m/z* (%): 285.02 (M^+^, 26.17), 104.08 (14.48), 160.06 (23.87), 146.01 (100.00).

*2-((4-Fluorophenoxy)methyl)-6,7-dimethoxy-4*H*-3,1-benzoxazin-4-one* (**3w**). ^1^H-NMR (400 MHz, CDCl_3_): δ 3.991 (d, *J* = 12 Hz, 6H, -OCH_3_), 4.963 (s, 2H, -OCH_2_-), 6.968 (d, *J* = 1.2 Hz, 2H, Ar-H), 7.005 (d, *J* = 4 Hz, 2H, Ar-H), 7.087 (s, 1H, Ar-H), 7.532 (s, 1H, Ar-H). IR (KBr) ν: 2920, 2806, 1670, 1611, 1507, 1433, 1222, 771 cm^−1^. EI-MS *m/z* (%): 331.03 (M^+^, 13.11), 220.04 (45.51), 191.99 (100.00).

*Step 3. Synthesis of* *2-phenoxymethyl–3*H*-quinazolin-4-ones* **4a**–**s**. 2-phenoxymethyl-3*H*-quinazolin-4-ones **4**a–s were synthesized according to the reported method [19]. Briefly, 2-phenoxyacetic-4*H*-3.1-benzoxazin-4-one (5 mmol) was heated with hydrazine hydrate (80% aqueous solution, 10 mL) or methylamine (33% methanol solution, 10 mL) in ethanol (5 mL) under reflux for 3 h. The clear and hot solution was filtered and then cooled down to room temperature. Crystals appeared and were collected to obtain the desired product with high purity. Molecular structures, melting points and yields are summarized in Table 5.

**Table 5:** Molecular structures, yields, and melting points of synthesized 2-phenoxy-3*H*-quinazolin-4-ones **4**.

No.	R^1^	R^2^	R^3^	R^4^	R^5^	R^6^	Appearance	Mp/°C	Yield/%
**4**a	Cl	H	H	H	Cl	NH_2_	white solid	191.8~193.3	70.1
**4**b	H	Cl	H	H	Cl	NH_2_	white solid	178.1~179.4	60.7
**4**c	H	H	Cl	H	Cl	NH_2_	white solid	129.5~131.6	59.1
**4**d	H	CH_3_	H	H	Cl	NH_2_	white solid	177.3~178.6	69.2
**4**e	H	OCH_3_	OCH_3_	H	Cl	NH_2_	white solid	202.8~204.1	81.3
**4**f	H	H	H	Cl	Cl	NH_2_	white solid	214.6~215.7	80.5
**4**g	Cl	H	H	Cl	Cl	NH_2_	white solid	201.5~202	76.4
**4**h	H	Cl	H	Cl	Cl	NH_2_	white solid	223.6~224.9	90.5
**4**i	H	H	Cl	Cl	Cl	NH_2_	white solid	129.1~131.3	85.6
**4**j	H	OCH_3_	OCH_3_	Cl	Cl	NH_2_	white solid	272.3~273.1	89.3
**4**k	Cl	H	H	H	Cl	CH_3_	white solid	184.9~185.7	65.1
**4**l	H	Cl	H	H	Cl	CH_3_	white solid	179.9~181.2	71.1
**4**m	H	H	Cl	H	Cl	CH_3_	white solid	202.4~203.9	67.3
**4**n	H	CH_3_	H	H	Cl	CH_3_	white solid	196.4~197.6	68.3
**4**o	H	OCH_3_	OCH_3_	H	Cl	CH_3_	white solid	201.8~202.7	70.9
**4**p	H	H	H	Cl	Cl	CH_3_	white solid	172.1~173.4	81.7
**4**q	H	Cl	H	Cl	Cl	CH_3_	white solid	240.1~241.8	84.6
**4**r	H	H	Cl	Cl	Cl	CH_3_	white solid	210.4~211.4	78.9
**4**s	H	OCH_3_	OCH_3_	Cl	Cl	CH_3_	white solid	243.9~245.2	91.3

*3-Amino-5-chloro-2-((4-chlorophenoxy)methyl)-3*H*-quinazolin-4-one* (**4a**). ^1^H-NMR (400 MHz, CDCl_3_): δ 4.411 (s, 2H, -CH_2_O-), 5.585 (s, 2H, N-NH_2_), 6.611(d, *J* = 7.6 Hz, 2H, Ar-H), 7.060 (d, *J* = 2.4 Hz, 2H, Ar-H), 7.496 (d, *J* = 8.0 Hz, 1H, Ar-H), 7.546 (d, *J* = 12.4 Hz, 2H, Ar-H). IR (KBr) ν: 3421, 2923, 1673, 1608, 1490, 1432, 1240, 771 cm^−1^. EI-MS *m/z* (%): 335.18 (M^+^, 67.58), 75.06 (82.84), 100.26 (66.29), 179.23 (100.00).

*3-Amino-6-chloro-2-((4-chlorophenoxy)methyl)-3*H*-quinazolin-4-one* (**4b**). ^1^H-NMR (600 MHz, CDCl_3_): δ 5.189 (s, 2H, -CH_2_O-), 5.293 (s, 2H, N-NH_2_), 6.995 (d, *J* = 8.4 Hz, 2H, Ar-H), 7.273 (d, *J* = 10.8 Hz, 2H, Ar-H), 7.699 (d, *J* = 9.6 Hz, 1H, Ar-H), 7.716 (d, *J* = 10.8Hz, 1H, Ar-H), 8.255 (s, 1H, Ar-H). IR (KBr) ν: 3435, 2987, 1747, 1681, 1537, 1494, 1242, 721 cm^−1^. EI-MS *m/z* (%): 335.06 (M^+^, 15.93), 208.11 (33.32), 335.06 (15.93), 179.06 (100.00).

*3-Amino-7-chloro-2-((4-chlorophenoxy)methyl)-3*H*-quinazolin-4-one *(**4c**). ^1^H-NMR (400 MHz, CDCl_3_): δ 5.168 (s, 2H, -CH_2_O-), 5.292 (s, 2H, N-NH_2_), 6.995 (d, *J* = 8.4 Hz, 2H, Ar-H), 7.272 (d, *J* = 8.4 Hz, 2H, Ar-H), 7.476 (d, *J* = 8.4 Hz, 1H, Ar-H), 7.749 (s, 1H, Ar-H), 8.109 (d, *J* = 4.4 Hz, 1H, Ar-H). IR (KBr) ν: 3480, 2983, 1679, 1550, 1493, 1245, 773 cm^−1^. EI-MS *m/z*, (%): 335.11 (M^+^, 19.55), 178.04 (33.81), 180.97 (31.33), 179.06 (100.00).

*3-Amino-2-((4-chlorophenoxy)methyl)-6-methyl-3*H*-quinazolin-4-one *(**4d**). ^1^H-NMR (400 MHz, CDCl_3_): δ 2.495 (s, 3H, Ar-CH_3_), 5.188 (s, 2H, -CH_2_O-), 5.278 (s, 2H, N-NH_2_),6.993 (d, *J* = 9.2 Hz, 2H, Ar-H), 7.254 (d, *J* = 8.4 Hz, 2H, Ar-H), 7.587 (d, *J* = 4.4 Hz, 1H, Ar-H), 7.629 (d, *J* = 4.8 Hz, 1H, Ar-H), 8.052 (s, 1H, Ar-H). IR (KBr) ν: 3466, 2964, 2924, 1747, 1676, 1550, 1494, 1244, 788 cm^−1^. EI-MS *m/z* (%): 315.07 (M^+^, 10.46), 188.11 (31.79), 159.02 (100.00).

*3-Amino-2-((4-chlorophenoxy)methyl)-6,7-dimethoxy-3*H*-quinazolin-4-one *(**4e**). ^1^H-NMR (400 MHz, CDCl_3_): δ 3.725 (s, 3H, N-CH_3_), 4.002 (s, 6H, -OCH_3_), 5.130 (s, 2H, -CH_2_O-), 5.285 (s, 2H, N-NH_2_), 7.013 (d, *J* = 8.8 Hz, 1H, Ar-H), 7.154 (s, 1H, Ar-H), 7.271 (d, *J* = 8.0 Hz, 1H, Ar-H), 7.573 (s, 1H, Ar-H). IR (KBr) ν: 3401, 2923, 2788, 1676, 1602, 1491, 1432, 1223, 773 cm^−1^. EI-MS *m/z* (%): 361.27 (M^+^, 7.07), 205.08 (69.78), 234.14 (100.00).

*3-Amino-2-((2,4-dichlorophenoxy)methyl)-3*H*-quinazolin-4-one *(**4f**). ^1^H-NMR (400 MHz, CDCl_3_): δ 5.373 (s, 2H, -CH_2_O-), 5.376 (s, 2H, -NH_2_), 7.132 (d, *J* = 4.8 Hz, 1H, Ar-H), 7.220 (d, *J* = 8.4 Hz, 1H, Ar-H), 7.386 (s, 1H, Ar-H), 7.556 (d, *J* = 6.8 Hz, 1H, Ar-H), 7.768~7.791 (m, 2H, Ar-H), 8.302 (d, *J* = 8.0, 1H, Ar-H). IR (KBr) ν: 3421, 2923, 1673, 1604, 1598, 1447, 1232, 771 cm^−1^. EI-MS *m/z* (%): 337.12 (M^+^, 1.62), 144.11 (30.64), 300.12 (72.15), 302.17 (16.80), 145.15 (100.00).

*3-Amino-5-chloro-2-((2,4-dichlorophenoxy)methyl)-3*H*-quinazolin-4-one *(**4g**). ^1^H-NMR (400 MHz, CDCl_3_): δ 5.295 (s, 2H, -CH_2_O-), 5.326 (s, 2H, -NH_2_), 7.191 (d, *J* = 8.2 Hz, 1H, Ar-H), 7.206 (d, *J* = 4.8 Hz, 1H, Ar-H), 7.248 (d, *J* = 11.2 Hz, 1H, Ar-H), 7.535 (s, 1H, Ar-H), 7.636 (d, *J* = 5.2 Hz, 2H, Ar-H), 8.302 (d, *J* = 8.0, 1H, Ar-H). IR (KBr) ν: 3490, 2992, 1676, 1608, 1479, 1432, 1233, 773 cm^−1^. EI-MS *m/z* (%): 369.06 (M^+^, 3.50), 178.09 (27.76), 336.13 (30.28), 179.05 (100.00). 

*3-Amino-6-chloro-2-((2,4-dichlorophenoxy)methyl)-3*H*-quinazolin-4-one* (**4h**). ^1^H-NMR (400 MHz, CDCl_3_): δ 5.342 (s, 2H, -CH_2_O-), 5.351 (s, 2H, -NH_2_), 7.115 (d, *J* = 2.0 Hz, 1H, Ar-H), 7.396 (d, *J* = 2.4 Hz, 1H, Ar-H), 7.697 (s, 1H, Ar-H), 7.708 (d, *J* = 2.0 Hz, 1H, Ar-H), 7.713 (d, *J* = 4.8 Hz, 1H, Ar-H), 8.268 (s, 1H, Ar-H). IR (KBr) ν: 3325, 3099, 1681, 1576, 1489,1253, 832 cm^−1^. EI-MS *m/z* (%): 368.90 (M^+^, 4.37), 208.10 (41.88), 334.0 (72.00), 366.00 (51.84), 179.01 (100.00).

*3-Amino-7-chloro-2-((2,4-dichlorophenoxy)methyl)-3*H*-quinazolin-4-one* (**4i**). ^1^H-NMR (600 MHz, CDCl_3_): δ 4.553 (s, 2H, -CH_2_O-), 4.599 (s, 2H, -NH_2_), 6.815 (d, *J* = 4.8 Hz, 1H, Ar-H), 6.837 (d, *J* = 2.4 Hz, 1H, Ar-H), 7.167 (s, 1H, Ar-H), 7.207 (d, *J* = 8.8 Hz, 1H, Ar-H), 7.228 (d, *J* = 5.2 Hz, 1H, Ar-H), 7.405 (s, 1H, Ar-H). ^13^C-NMR (CDCl_3_) δ: 69.21, 117.26, 118.65, 122.89, 124.09, 124.49, 127.62, 128.07, 130.31, 131.39, 139.07, 147.78, 151.98, 160.11,164.05. IR (KBr) ν: 3395, 2929, 1673, 1610, 1501, 1483, 1251,771 cm^−1^. EI-MS *m/z*(%):368.99 (M^+^, 2.58), 179.07 (86.13), 181.13 (31.11), 334.14 (50.05), 336.15 (21.99), 162.02 (100.00).

*3-Amino-2-((2,4-dichlorophenoxy)methyl)-6,7-dimethoxy-3*H*-quinazolin-4-one *(**4j**). ^1^H-NMR (400 MHz, CDCl_3_): δ 3.871 (s, 3H, -OCH_3_), 3.886 (s, 3H, -OCH_3_), 5.434 (s, 2H, -CH_2_O-), 5.638 (s, 2H, -NH_2_), 7.006 (s, 1H, Ar-H), 7.192 (d, *J* = 8.8 Hz, 1H, Ar-H), 7.320 (d, *J* = 2.4 Hz, 1H, Ar-H), 7.445 (s, 1H, Ar-H), 7.618 (s, 1H, Ar-H). IR (KBr) ν: 3390, 2927, 1673, 1610, 1599, 1501, 1253, 806 cm^−1^. EI-MS *m/z* (%): 395.04 (M^+^, 6.84), 205.05 (65.95), 234.06 (100.00).

*5-Chloro-2-((4-chlorophenoxy)methyl)-3-methyl-3*H*-quinazolin-4-one *(**4k**). ^1^H-NMR (400 MHz, CDCl_3_): δ 3.684 (s, 3H, N-CH_3_), 5.124 (s, 2H, -CH_2_O-), 6.986 (d, *J* = 8.8 Hz, 2H, Ar-H), 7.270 (d, *J* = 8.0 Hz, 2H, Ar-H), 7.507 (d, *J* = 9.2 Hz, 1H, Ar-H), 7.603 (d, *J* = 3.2 Hz, 1H, Ar-H). IR (KBr) ν: 2990, 2923, 1675, 1600, 1479, 1425, 1245, 785 cm^−1^. EI-MS *m/z* (%): 334.00 (M^+^, 6.00), 168.06 (20.78), 209.01 (18.86), 166.29 (100.00). 

*6-Chloro-2-((4-chlorophenoxy)methyl)-3-methyl-3*H*-quinazolin-4-one *(**4l**). ^1^H-NMR (600 MHz, CDCl_3_): δ 3.733 (s, 3H, N-CH_3_), 5.155 (s, 2H, -CH_2_O-), 6.995 (d, *J* = 9.0 Hz, 2H, Ar-H), 7.272 (d, *J* = 9.0 Hz, 2H, Ar-H), 7.667 (d, *J* = 9.0 Hz, 1H, Ar-H), 7.702 (d, *J* = 9.0 Hz, 1H, Ar-H), 8.260 (s, 1H, Ar-H). IR (KBr) ν: 3096, 2924, 1677, 1602, 1481, 1245, 785 cm^−1^. EI-MS *m/z* (%): 334.12 (M^+^, 13.55), 166.03 (67.05), 207.04 (100.00).

*7-Chloro-2-((4-chlorophenoxy)methyl)-3-methyl-3*H*-quinazolin-4-one *(**4m**). ^1^H-NMR (400 MHz, CDCl_3_): δ 3.720 (s, 3H, N-CH_3_), 5.147 (s, 2H, -CH_2_O-), 6.993 (d, *J* = 9.0 Hz, 2H, Ar-H), 7.267 (d, *J* = 8.8 Hz, 2H, Ar-H), 7.459 (d, *J* = 6.8 Hz, 1H, Ar-H), 7.698 (s, 1H, Ar-H), 8.213 (d, *J* = 4.8 Hz, 1H, Ar-H). IR (KBr) ν: 3038, 2923, 1674, 1596, 1482, 1421, 1240, 781 cm^−1^. EI-MS *m/z* (%): 334.09 (M^+^, 11.78), 167.96 (24.42), 209.17 (19.90), 166.00(100.00).

*2-((4-Chlorophenoxy)methyl)-3,6-dimethyl-3*H*-quinazolin-4-one *(**4n**). ^1^H-NMR (400 MHz, CDCl_3_): δ 2.498 (s, 3H, Ar, -CH_3_), 3.726 (s, 3H, N-CH_3_), 5.152 (s, 2H, -CH_2_O-), 6.997 (d, *J* = 5.2 Hz, 2H, Ar-H), 7.259 (d, *J* = 9.2 Hz, 2H, Ar-H), 7.585 (d, *J* = 2.0 Hz, 1H, Ar-H), 7.599 (d, *J* = 4.0 Hz, 1H, Ar-H), 8.082 (s, 1H, Ar-H). IR (KBr) ν: 3080, 2924, 1674, 1599, 1490, 1423, 1241, 782 cm^−1^. EI-MS *m/z* (%): 314.23 (M^+^, 16.06), 146.15 (79.66), 158.07 (77.83), 187.13 (100.00).

*2-((4-Chlorophenoxy)methyl)-6,7-dimethoxy-3-methyl-3*H*-quinazolin-4-one *(**4o**). ^1^H-NMR (400 MHz, CDCl_3_): δ 4.007 (s, 3H, -OCH_3_), 4.035 (s, 3H, -OCH_3_), 5.176 (s,2H, -CH_2_O-), 6.992 (d, *J* = 4.8 Hz, 1H, Ar-H), 7.091 (s, 1H, Ar-H), 7.265 (d, *J* = 8.8 Hz, 1H, Ar-H), 7.602 (s, 1H, Ar-H). IR (KBr) ν: 3042, 2900, 1675, 1598, 1487, 1424, 1240, 816 cm^−1^. EI-MS *m/z* (%): 360.18 (M^+^, 6.41), 192.11 (19.88), 204.11 (18.44), 233.16 (100.00).

*2-((2,4-Dichlorophenoxy)methyl)-3-methy-3*H*-quinazolin-4-one* (**4p**). ^1^H-NMR (400 MHz, CDCl_3_): δ 3.806 (s, 3H, N-CH_3_), 5.264 (s, 2H, -CH_2_O-), 7.176 (d, *J* = 4.8 Hz, 1H, Ar-H), 7.190 (d, *J* = 2.0 Hz, 1H, Ar-H), 7.389 (s, 1H, Ar-H), 7.329~7.533 (m, 1H, Ar-H), 7.707 (d, *J* = 8.0 Hz, 1H, Ar-H), 7.755~7.775 (m, 1H, Ar-H), 8.305 (d, *J* = 8.0 Hz, 1H, Ar-H). IR (Br) ν: 2984, 1676, 1570, 1476, 1251, 773 cm^−1^. EI-MS *m/z* (%): 334.12 (M^+^, 5.76), 144.08 (35.49), 173.09 (51.15), 299.09 (76.34), 132.05 (100.00).

*6-Chloro-2-((2,4-dichlorophenoxy)methyl)-3-methyl-3*H*-quinazolin-4-one* (**4q**). ^1^H-NMR (400 MHz, CDCl_3_): δ 3.798 (s, 3H, N-CH_3_), 5.235 (s, 2H, -CH_2_O-), 7.016 (d, *J* = 4.8 Hz, 1H, Ar-H), 7.112 (d, *J* = 8.4 Hz, 1H, Ar-H), 7.233 (d, *J* = 4.8 Hz, 1H, Ar-H), 7.341 (s, 1H, Ar-H), 7.418 (d, *J* = 8.8 Hz, 1H, Ar-H), 7.421 (s, 1H, Ar-H). IR (KBr) ν: 2970, 1675, 1602, 1480, 1426, 1290, 788 cm^−1^. EI-MS *m/z* (%): 370.10 (M^+^, 5.19), 178.09 (55.72), 333.03 (51.79), 207.09 (100.00).

*7-Chloro-2-((2,4-dichlorophenoxy)methyl)-3-methyl-3*H*-quinazolin-4-one* (**4r**). ^1^H-NMR (400 MHz, CDCl_3_): δ 3.789 (s, 3H, N-CH_3_), 5.237 (s, 2H, -CH_2_O-), 7.145 (d, *J* = 9.0 Hz, 1H, Ar-H), 7.206 (d, *J* = 9.0 Hz, 1H, Ar-H), 7.394 (s, 1H, Ar-H), 7.472 (d, *J* = 8.4 Hz, 1H, Ar-H), 7.694 (s, 1H, Ar-H), 8.225 (d, *J* = 8.4 Hz, 1H, Ar-H). IR (KBr) ν: 2924, 2854, 1675, 1602, 1480, 1426, 1290, 788 cm^−1^. EI-MS *m/z* (%): 367.79 (M^+^, 2.31), 207.08 (25.72), 333.05 (37.41), 166.13 (100.00).

*2-((2,4-Dichlorophenoxy)methyl)-6,7-dimethoxy-3-methyl-3*H*-quinazolin-4-one* (**4s**) ^1^H-NMR (400 MHz, CDCl_3_): δ 3.799 (s, 3H, N-CH_3_), 4.007(s, 6H, -OCH_3_), 5.229 (s, 2H, -CH_2_O-), 7.006 (s, 1H, Ar-H), 7.182 (d, *J* = 4.8 Hz, 1H, Ar-H), 7.221 (d, *J* = 2.0 Hz, 1H, Ar-H), 7.341 (s, 1H, Ar-H), 7.418 (s, 1H, Ar-H), 7.421 (s, 1H, Ar-H). IR (KBr) ν: 306, 2835, 1673, 1637, 1497, 1245, 761 cm^−1^. EI-MS *m/z* (%): 394.07 (M^+^, 23.01), 192.08 (17.63), 204.11 (15.13), 233.11 (100.00).

### 3.3. Herbicidal Acitivity Evaluation

The herbicidal activity was examined for the growth inhibition effect of the dicotyledon rape *Brassica napus* and the monocotyledon barnyard grass *Echinochloa crusgalli*, using the standard Petri dish test and the pre-emergence treatment procedure [20]. Briefly, the compounds to be tested were dissolved in DMF and emulsified with Tween 80, and the solutions were diluted with water to the concentrations of 10 mg/mL and 1 mg/mL, respectively. Nine mL of the solution was added in a Petri dish of 9 cm diameter and two pieces of filter paper were placed on the dish bottom. Twenty seeds each of rape and barnyard grass were placed on the filter paper. The covered Petri dish was transferred into an artificial climate incubator (MGC-400H, Shantou Keyi Co., Ltd., China), where the conditions were controlled at temperature 23 ± 1 °C, humidity 60 ± 5%, light intensity 10 Klux, and photoperiod 12 h/day. The incubation was continued for 5 days, then the lengths of 10 roots and 10 stems for each type of plant were measured and the mean values were calculated. The percent inhibitory ratios were calculated relative to control plants exposing to distilled water instead of the compound solution.

### 3.4. Molecular Docking

The complex crystal data of TIR1/NAA (PDB ID: 2P1O) was chosen and prepared for the docking receptor by extracting all water molecules and the bound ligand NAA. The ligand files of selected compounds were prepared using the energy-minimized conformations. The compiled config file was executed in Vina and the docking conformation with the lowest binding energy was chosen for the comparison. Docking was performed using the default docking parameters in Vina software following the introduction of the designer Dr. Oleg Trott in the Molecular Graphics Lab at the Scripps Research Institute [15]. The visualization and comparison of the docking results were realized using the tool MGLTools 1.5.4. In order to validate the credibility of the docking procedure, NAA was also docked and superimposed with its crystal conformation in the binding site of TIR1 receptor. 

## 4. Conclusions

In summary, we have developed a facile and convenient strategy for the synthesis of series of novel 4*H*-3,1-benzoxazin-4-ones and 3*H*-benzoquinazolin-4-ones with (un)substituted phenoxymethyl moieties in good to excellent yields by simply using the one-pot *N*-acylation/ring closure reaction of anthranilic acids and 2-phenoxyacetyl chlorides and subsequent substitution with amino derivatives. The herbicidal tests showed that most of the target compounds had high herbicidal activities against the root growth of dicotyledonous rape, and the preliminary structure-activity relationships of these new compounds are discussed. The active substructure analysis of the title compounds and the typical inhibitory phenotype of hormone herbicides implicate their target protein is the auxin receptor TIR1. Molecular docking experiments showed that the selected compounds **3**o and **4**i can be fitted well in the active site with relatively more negative affinities than the typical hormone herbicide 2,4-D, although their inhibitory efficacies are less than 2,4-D. Further investigation on the structural optimization of these compounds for improving the herbicidal activity and the *in vitro* activity measurement against the native TIR1 are possible, and is in progress in this laboratory.
